# Preadolescent and Adolescent Victims of Cyber Victimization in Tunisia

**DOI:** 10.1192/j.eurpsy.2024.956

**Published:** 2024-08-27

**Authors:** A. Touiti, W. Askri, S. Halayem, A. Bouden

**Affiliations:** ^1^Children and Adolescent Psychiatric Department, Razi Hospital, La Manouba; ^2^Faculty of Medicine of Tunis, Tunis El Manar University, Tunis, Tunisia

## Abstract

**Introduction:**

Nowadays children and adolescents are exposed to cyber victimization.This modern form of aggressive behavior has a negative impact on the psychological of victims,self esteem, and social interaction

**Objectives:**

To investigate the relation between cybervictimization and depression in tunisian preadolescents and adolescents

**Methods:**

The Arabic validated version of the “cyberbullying assessment instrument” was distributed through social media groups of preadolescent and adolescents in Tunisia.The participants were also invited to answer items about social and demographic characteristics.The participation was voluntary,without confidential data.

**Results:**

Fifty four preadolescent and adolescent aged between 9 and 16 years old have participated. The average age was 12.4 years old. 64% of participants were girls.More than 80% of children have their own smartphone and a personal count on social media.Among those respondents,12 (22.2%) reported being cyberbullied at least once in the year.the children most likely to be bullied were girl aged between 9 and 12 years old with a poor socioeconomic level.low self esteem, depressive symptoms, anxiety symptoms are associated with cyber victimization.

**Conclusions:**

The level of cyber victimization among preadolescents and adolescents is underestimated. Psychiatric disorder associated to this phenomena have to be considered in order to develop strategies and intervention to reduce the cyberbullying among vulnerable population.

**Disclosure of Interest:**

None Declared

